# Combination BMSC and Niaspan Treatment of Stroke Enhances White Matter Remodeling and Synaptic Protein Expression in Diabetic Rats

**DOI:** 10.3390/ijms141122221

**Published:** 2013-11-11

**Authors:** Xinchun Ye, Tao Yan, Michael Chopp, Alex Zacharek, Ruizhuo Ning, Poornima Venkat, Cynthia Roberts, Jieli Chen

**Affiliations:** 1 Department of Neurology, the Affiliated Hospital of Xuzhou Medical College, Xuzhou 221002, China; E-Mail: xinchunye@gmail.com; 2 Department of Neurology, Henry Ford Hospital, Detroit, MI 48202, USA; E-Mails: yantao78@hotmail.com (T.Y.); mchopp1@hfhs.org (M.C.); alexzacharek@gmail.com (A.Z.); nsrzn@neuro.hfh.edu (R.N.); pvenkat@oakland.edu (P.V.); cindi@neuro.hfh.edu (C.R.); 3 Department of Neurology, Tianjin Medical University General Hospital, Tianjin Neurological Institute, Tianjin 300052, China; 4 Department of Physics, Oakland University, Rochester, MI 48309, USA

**Keywords:** type-one diabetic rats, stroke, bone marrow stromal cells, Niaspan, white matter remodeling, synaptic plasticity

## Abstract

**Objective:**

White matter remodeling plays an important role in neurological recovery after stroke. Bone marrow stromal cells (BMSCs) and Niaspan, an agent which increases high density lipoprotein (HDL), each induces neurorestorative effects and promotes white matter remodeling after stroke in non-diabetic rats. In this study, we test whether combination of BMSCs with Niaspan induces an enhanced white matter remodeling in the ischemic brain of diabetic rats.

**Research design and methods:**

Type-1 diabetes (T1DM) rats were subjected to transient middle cerebral artery occlusion (MCAo) and treated with or without BMSCs; Niaspan; and the combination of BMSCs + Niaspan daily for 14 days after MCAo. Immunostaining for white matter remodeling and synaptic protein expression including NG2; CNPase; BS (Bielschowsky silver); LFB (luxol fast blue); Synaptophysin and SMI-31 immunostaining were performed.

**Results:**

BMSC monotherapy did not regulate NG2 and CNPase expression compared to T1DM control rats. Both, combination of BMSCs + Niaspan treatment, and Niaspan monotherapy significantly increase NG2 and CNPase expression compared to T1DM control. While combination BMSC+Niaspan, BMSC monotherapy and Niaspan monotherapy groups all increase BS, LFB, synaptophysin, and SMI-31 expression in the ischemic brain compared to T1DM-MCAo control. In addition, the combination treatment significantly enhances LFB, SMI-31, and Synaptophysin expression compared to BMSC monotherapy.

**Conclusions:**

Combination treatment of stroke with BMSCs and Niaspan in T1DM rats increases white matter remodeling and additively increases BMSC monotherapy induced myelination and synaptic plasticity after stroke in T1DM rats.

## 1. Introduction

Diabetes mellitus (DM) is associated with both microvascular and macrovascular disease [1] and is a major health problem known to substantially elevate the risk of occurrence and recurrence of ischemic stroke [2–5]. Functional outcomes and fatality rates are significantly worse in the diabetic stroke population [6,7]. DM patients face greater residual neurological and functional disability when compared with non-diabetic subjects [3]. Our previous data have indicated that treatment with bone marrow stromal cells (BMSCs) improves functional outcome after stroke in non-diabetic rats [8,9]. BMSCs pass through the blood brain barrier (BBB) and selectively target damaged brain, secreting growth factors [10,11], and inducing parenchymal cell secretion of growth factors, increase synaptogenesis [12], and thereby improving functional recovery [8,13,14].

Niacin (nicotinic acid) is one of the most effective medications in clinical use for increasing high-density lipoprotein (HDL) [15,16]. Niaspan is a prolonged release formulation of niacin and is safely used in patients with diabetes [17]. Treatment of stroke with Niaspan significantly increases Angiopoietin-1 (Ang1) expression in ischemic brain which promotes vascular stabilization [18] and decreases brain hemorrhage and BBB leakage in T1DM rats [16,19] and regulates angiogenesis as well as improves functional outcome after stroke [15,16]. White matter remodeling and synaptogenesis play an important role in neurorestorative effects after stroke [20,21]. In this study, we tested whether combination BMSCs with Niaspan enhances white matter remodeling and synaptic protein expression after stroke in T1DM rats.

## 2. Results

### 2.1. Combination BMSCs and Niaspan Treatment of Stroke Increases White Matter (WM) Remodeling

**Figure 1:**
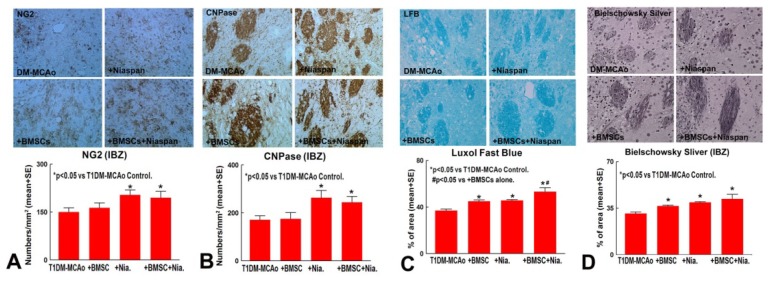

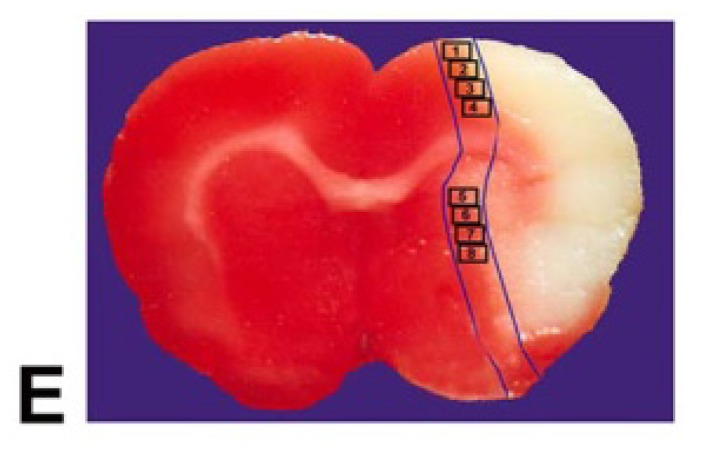
Oligodendrocyte progenitor cells (OPC), Oligodendrocytes (OLs), and myelination measurements. Treatment of stroke with BMSCs, Niaspan and combination BMSCs+Niaspan all significantly increased myelin and axon density as identified by LFB (**C**, *p* <0.05) and Bielschowsky Silver (**D**, *p* < 0.05) staining compared to T1DM-MCAo control. Niaspan and combination BMSCs+Niaspan treatment both significantly increased OPCs and OLs number as identified by NG2 (**A**, *p* <0.05) and CNPase (**B**, *p* < 0.05) staining expression in the ischemic brain compared to T1DM-MCAo control. Combination treatment additively increases myelin density (LFB) compared to BMSCs monotherapy rats (*p* <0.05). (**E**): shows the immunostaining measurement area in the ischemic border area. Group number in T1DM-MCAo: *n* = 8; Niaspan alone: *n* = 10; BMSCs alone: *n* = 8; BMSCs+Niaspan: *n* = 7. * *p* < 0.05 *vs.* T1DM-MCAo control.

OPCs (Oligodendrocyte progenitor cells) and OLs (Oligodendrocytes) are needed for myelination, while myelination is critical for axonal function. OPCs contribute to myelin maintenance and repair by generating new OLs as a source of remyelination and repair after stroke [22]. To test whether combination treatment regulates white matter remodeling, CNPase (OL marker), NG2 (OPC marker), Luxol Fast Blue (LFB, myelin marker), and Bielschowsky Silver (BS, axon marker) staining were performed. Figure 1A–B shows that Niaspan monotherapy and combination therapy of BMSCs + Niaspan both significantly increased OPCs (*p* < 0.05, Figure 1A) and OLs (*p* < 0.05, Figure 1B) number in the ischemic boundary zone compared to non-treatment control (*p* < 0.05), while BMSCs monotherapy did not increase NG2 and CNPase expression compared to T1DM-MCAo control (*p* > 0.05). Figure 1C,D show that treatment of stroke with BMSCs alone, Niaspan alone, and combination of BMSCs + Niaspan, all significantly increased myelin and axon density as indicated by an increased expression in immunostaining with LFB (*p* < 0.05, Figure 1C) and BS (*p* < 0.05, Figure 1D) compared to T1DM-MCAo control rats. The combination treatment also induces an additive effect on increasing LFB density (*p* < 0.05), but not in NG2 and CNPase (*p* > 0.05) expression in the ischemic brain when compared to BMSCs monotherapy. The data indicated that combination treatment enhances BMSC induced myelination in the ischemic brain.

### 2.2. Combination BMSCs and Niaspan Treatment of Stroke Additively Enhances Synaptic Protein Expression

**Figure 2:**
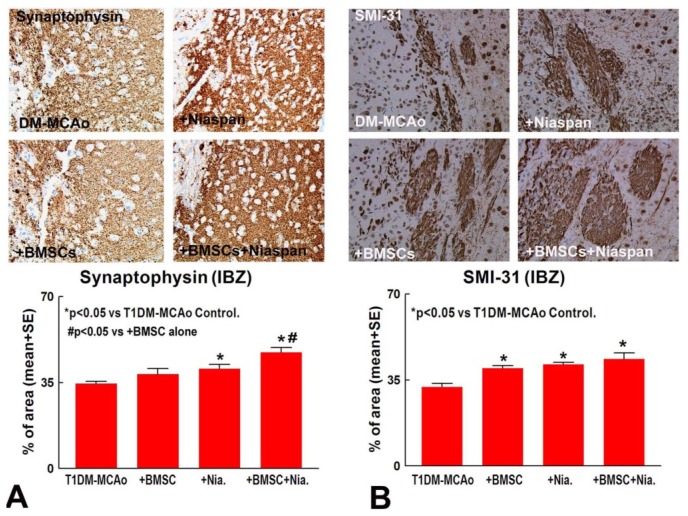
Treatment of stroke with BMSCs, Niaspan and combination BMSCs+Niaspan all significantly increased SMI-31 (**A**, *p* < 0.05) expression compared to T1DM-MCAo control. Niaspan and combination BMSCs+Niaspan treatment both increased Synaptophysin (**B**, *p* < 0.05) expression in the ischemic brain compared to T1DM-MCAo control. Combination treatment additively increases Synaptophysin expression compared to BMSCs monotherapy rats (*p* < 0.05). Group number in T1DM-MCAo: *n* = 8; Niaspan-alone: *n* = 10; BMSCs-alone: *n* = 8; BMSCs + Niaspan: *n* = 7. * *p* < 0.05 *vs.* T1DM-MCAo control.

To test whether combination treatment regulates synaptic protein expression, Synaptophysin and SMI-31 (a pan-axonal neurofilament marker) immunostaining were performed. Figure 2A–B show that Niaspan monotherapy and combination of BMSC + Niaspan treatment of stroke significantly increased Synaptophysin (*p* < 0.05, Figure 2A) and SMI31 (*p* < 0.05, Figure 2B) expression compared to T1DM-MCAo control. (T1DM-MCAo control *n* = 8; Niaspan *n* = 10; BMSCs *n* = 8; BMSCs + Niaspan *n* = 7). The combination treatment induces an additive effect on increasing synaptic protein Synaptophysin expression when compared to BMSCs monotherapy rats (*p* < 0.05). The data indicated that combination treatment enhances synaptic protein expression in the ischemic brain.

### 2.3. Combination BMSCs and Niaspan Treatment of Stroke Increases CNPase, Synaptophysin, and SMI-31 Expression in the Ischemic Brain

**Figure 3:**
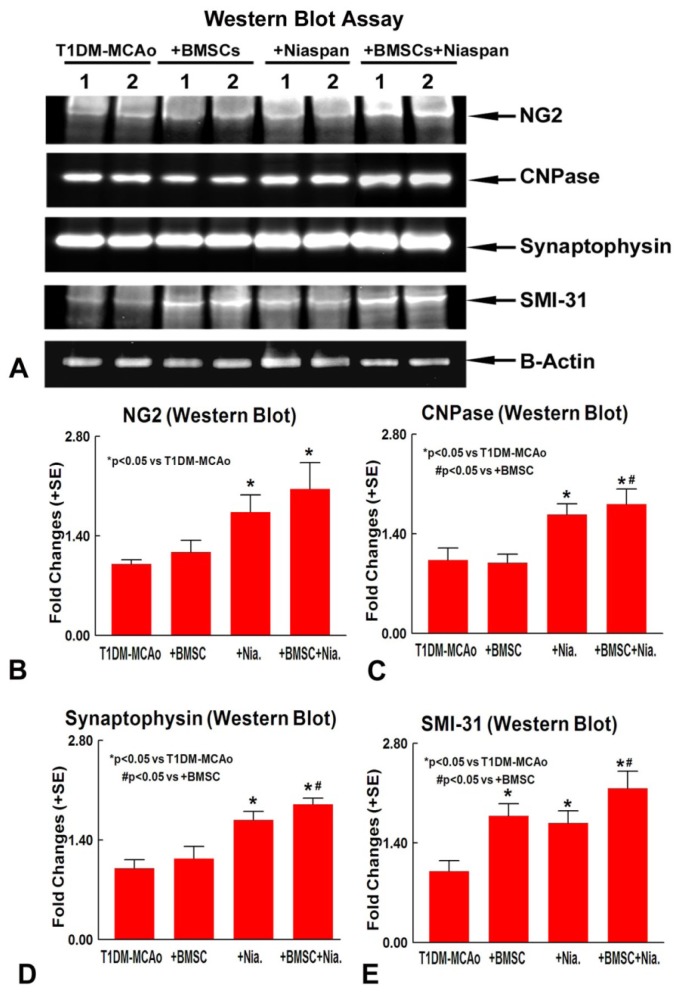
Western Blot assay. (**A**) Western blot assay for NG2, CNPase, Synaptophysin and SMI-31; (**B**–**E**) Western blot quantitative data for NG2 (**B**); CNPase (**C**); Synaptophysin (**D**) and SMI-31 (**E**). *n* = 4/group.

To confirm the immunostaining data, Western blot assay was employed to measure NG2, CNPase, Synaptophysin, and MSI-31 protein expression. Figure 3 shows that BMSCs monotherapy did not increase NG2 (A and B), CNPase (A and C), and Synaptophysin (A and D) expression, but significantly increased SMI-31 (A and E) protein expression compared to T1DM-MCAo control. Niaspan alone, and combination of BMSC+Niaspan, treatment all significantly increased NG2, CNPase, Synaptophysin, and SMI-31 (A–E) expression compared to T1DM-MCAo control; While combination treatment also additively increased CNPase, Synaptophysin and SMI-31 (C–E) expression when compared to BMSC monotherapy. The data indicate that combination treatment increases OL and synaptic plasticity compared to BMSC monotherapy.

## 3. Discussion

Our previous study show that found that combination BMSCs and Niaspan treatment has a trend of decrease mortality rate (*p* = 0.07), but did not significantly decrease lesion volume and improve functional outcome after stroke when compared to non-treatment control or monotherapy (*p* > 0.05) [23]. In this study, we have demonstrated that combination therapy of BMSCs + Niaspan in diabetic rats significantly increased OPCs and OLs number, increased axonal and myelin density in the ischemic brain compared to non-treatment T1DM control. Our data also demonstrate that combination treatment of stroke in diabetic rats significantly increased Synaptophysin and SMI31 expression compared to non-treatment T1DM control. The combination of BMSCs and Niaspan treatment of stroke in T1DM rats additively enhances myelin density and synaptic protein Synaptophysin expression compared to BMSCs monotherapy.

White matter remodeling and synaptic plasticity play an important role in long-term functional outcome. Oligodendrocytes are the myelin-forming glial cells in the adult brain and are highly vulnerable to ischemic stroke [24]. Non-myelinating OPCs in the corpus callosum and the striatum can be induced to differentiate into OLs after ischemic stroke [25]. Damage to OLs and OPCs causes loss of myelin synthesis and interruption of proper axonal function. Hence, even if we protect neurons in gray matter, loss of myelin and axonal integrity would interfere with neuronal connectivity and function [26]. In the present study, we have shown that the most important benefit of using Niaspan and BMSCs combination therapy is its additive effect upon white matter remodeling. We found that BMSC treatment did not significantly increase NG2 and CNPase expression in the ischemic brain, while Niaspan and combination treatment significantly increased NG2 and CNPase expression compared to T1DM-MCAo control. However, significant differences in the NG2 (OPC marker) and CNPase (OLs marker) expression between BMSCs+Niaspan *versus* the Niaspan monotherapy group were not detected. In addition, we found that the BMSC and Niaspan monotherapy, respectively, and combination treatment all significantly increased LFB, Synaptophysin, and SMI-31 expression, and the combination treatment induces additive effects on the regulation of LFB, Synaptophysin and SMI-31 expression were measured by immunostaining and Western blot assays. The data indicate that combination of BMSCs+Niaspan increases myelination and synaptic protein expression, but did not increase OPC density when compared to BMSC monotherapy. Additional investigations are warranted on the mechanisms by which combination treatment provides a beneficial effect on the myelination and synaptic plasticity.

Synaptic plasticity enhances functional recovery by axonal and dendritic regeneration and reorganization within cortical motor areas [27]. Axonal reorganization via sprouting of nearby axons has been implicated in spontaneous recovery after infarction in rodent models of stroke [28]. Niaspan monotherapy and combination therapy with BMSCs substantially increased Synaptophysin expression in the ischemic brain as well as increased SMI expression compared to T1DM-MCAo control. The combination treatment of stroke significantly induces an additive increase in synaptic plasticity identified by increased Synaptophysin expression in the ischemic brain when compared to the monotherapy with BMSCs. Enhanced synaptic plasticity may be beneficial as Synaptogenesis promotes neurorestorative effects and enhances functional outcome post stroke [20]. Increasing the expression of LFB, BS, Synaptophysin, and SMI31 in T1DM-MCAo rats by combination treatment may contribute to improvement of white matter remodeling and synaptic plasticity in the ischemic brain, which may enhance functional outcome after stroke in diabetic rats.

There are a few limitations to this study. Long term plasticity is mediated by synaptogenesis, axonal and dendrite plasticity that accounts for creation of new synapses and pathways, while short term plasticity is mediated by activation of parallel and silent pathways that can restore impaired functions [29,30]. White matter remodeling could be a sequel to a number of mechanisms and our study did not investigate the factors that regulate it. As a caveat, in this study, we only sacrificed animals at 14 days after MCAo and measured the white matter changes at this time point. Measurements at additional time points are warranted.

## 4. Experimental Section

All experiments were conducted in accordance with the standards and procedures of the American Council on Animal Care and Institutional Animal Care and Use Committee of Henry Ford Health System.

### 4.1. Diabetes Induction

Adult Male Wistar rats (225–250 g) purchased from Charles River (Wilmington, MA, USA) were used. Diabetes was induced by a single intraperitoneal injection of streptozotocin (STZ, 60 mg/kg, Sigma Chemical Co., St. Louis, MO, USA) to rats [31]. The fasting blood glucose level was tested by using a glucose analyzer (Accu-Chek Compact System; Roche Diagnostics, Indianapolis, IN, USA). Animals were subjected middle cerebral artery occlusion (MCAo) 2 weeks after diabetes induction (fasting blood glucose >300 mg/dL) [31].

### 4.2. BMSCs Culture

Rat bone marrow cells were isolated from the tibia and femur of 8-week old male Wistar rats, as described previously [13]. Briefly, fresh bone marrow (BM) was harvested aseptically from tibias and femurs, by inserting a 21-gauge needle connected to a 1-ml syringe into the shaft of the bone and flushing with 1× PBS. The resulting BM was dissociated into a milky homogenous single-cell suspension. 0.84% NHCl was used to remove red blood cells. Following, 2 × 10^6^ nucleated cells were seeded into each tissue culture flask in Hyclone MEM Alpha Modification media (Thermo Scientific) supplemented with 20% fetal bovine serum. After 72 h of incubation, non-adherent cells were removed and adherent cells were collected and resuspended in fresh medium in new flasks. In our, and other, previous studies to identify BMSCs, flow cytometry analysis was performed on cells from passages 3–5. CD29 and CD44 are BMSC surface markers, while CD14 exists in monocytes and macrophages and CD34 is a hematopoietic stem cell marker. In our previous studies, we have found that 95.3% and 98.1% of the cultured cells expressed CD29 and CD44, respectively [32]. While CD14 and CD34 positive rates in cultured cells were 1.9% and 1.3%. Therefore, high purity BMSCs can be harvested simply and effectively by their adherent characteristics. The present study used the same BMSC culture methods, and as such, flow cytometry analysis was not repeated.

### 4.3. MCAo Model and Experiment Groups

T1DM rats were initially anesthetized with 3.5% isoflurane and maintained with 1.0% to 2.0% isoflurane in 70% N_2_O and 30% O_2_ by a face mask. Rectal temperature was maintained at 37 °C throughout the surgical procedure by means of a feedback-regulated water heating system. A 4-0 nylon suture with its tip rounded by heating near a flame was inserted into the external carotid artery (ECA) through a small puncture. The length of nylon suture, determined according to the animal’s weight, was gently advanced from the ECA into the lumen of the internal carotid artery (ICA) until the suture blocked the origin of the middle cerebral artery (MCA) as previously described [8]. After 2 hours of MCAo, restoration of blood flow was performed by withdrawal of the filament. Rats were randomized and assigned to different groups and were treated starting 24 h after MCAo with: (1) PBS (Phosphate Buffered Saline) as vehicle control (*n* = 8); (2) BMSCs (5 × 10^6^) alone (*n* = 8) via tail vein injection; (3) Niaspan (40 mg/kg, Kos Pharmaceuticals, Inc. Cranbury, NJ, USA; dissolved in saline) alone orally daily for 14 days (*n* = 10); (4) BMSCs (5 × 10^6^) (iv injection) + Niaspan (40 mg/kg, orally daily for 14 days) combination treatment (*n* = 7). Rats were sacrificed at 14 days after MCAo.

### 4.4. Immunohistochemical Assessment

All brains were fixed by transcardial perfusion with saline, followed by perfusion and immersion in 4% paraformaldehyde then embedded in paraffin. A standard block was obtained from the center of the lesion (bregma −1 mm ~ +1 mm). A series of 6 μm thick sections was cut from the block. Every 10^th^ coronal section, for a total 5 sections, was used for immunohistochemical staining. After deparafinizing, brain sections were boiled for 10 min in antigen retrieval citrate buffer (pH6). After blocking in normal serum, brain sections were treated with the primary antibodies against NG2 (oligodendrocyte progenitor cell marker, 1:400; Millipore, Billerica, MA, USA), CNPase (a prenylated myelin protein, 1:200; Millipore, Billerica, MA, USA), Synaptophysin (monoclonal antibody; dilution 1:500, Millipore, MA, USA, and SMI-31 (a pan-axonal neurofilament marker, Neurofilaments, phosphorylated monoclonal antibody, 1:1000, Covance, Emeryville, CA, USA) overnight. Diaminobenzidine was then used as a chromogen for light microscopy. Counterstaining of sections by hematoxylin was also performed. Bielschowsky Silver Staining method (Theory and Practice of Histotechnology by Sheehan & Hrapchak, second edition) was used to stain axons. Briefly, for Bielschowsky staining, slides were placed in 20% silver nitrate in the dark, then ammonium hydroxide was added, and the slides were then treated with NaOH and sodium thiosulfate. Luxol Fast Blue was used to stain myelin [33]. Negative control sections from each animal received identical preparations for immunohistochemical staining, except that primary antibodies were omitted. The immunostaining analysis was performed by an investigator blinded to the experimental groups. Fields of interest were selected in the ischemic border zone, and 8 fields in each slide were chosen to analyze; please see Figure 1E.

### 4.5. Quantitation Measurements of NG2, CNPase, Synaptophysin, BS, and LFB

For quantitative measurements of NG2, CNPase, Synaptophysin, BS, and LFB, five slides from each brain, with each slide containing 8 fields of view from the ischemic border zone (IBZ, Figure 1E) were digitized under a 20× objective (Olympus BX40) using a 3-CCD color video camera (Sony DXC-970MD) interfaced with an MCID image analysis system (Imaging Research, St. Catharines, Canada). The ischemic border zone is defined as the area surrounding the lesion. Positive areas of immunoreactive cells were measured in the IBZ (Figure 1E). Data were analyzed in a blinded manner.

### 4.6. Western Blot Assay

An additional set of animals (*N* = 4/group) were sacrificed at 14 days after MCAo. Brain tissue extract was isolated from ischemic brain border area using Trizol (Invitrogen, Grand Island, NY, USA), following standard protocol. Protein concentration was measured using the BCA (Thermo Scientific, Waltham, MA, USA) kit. 40 ug of protein/lane in a 10% SDS PAGE precast gel (Invitrogen, Grand Island, NY, USA). Gel was transferred using an iBlot transfer system (Invitrogen, Grand Island, NY, USA) following standard protocol. Nitrocellulose membrane was blocked in 2% I-Block (Applied Biosystems, Foster City, NY, USA) in 1× TBS-T for one hour, and then either CNPase (1:5000, Millipore, Billerica, MA, USA), NG2 (1:1000, Millipore, Billerica, MA, USA), SMI-31 (1:1000, Covance, Emeryville, CA, USA), or Synaptophysin (1:2000, Millipore, Waltham, MA, USA) primary antibodies were added in 2% I-Block in TBS-T, and incubated on a shaker overnight at 4 °C. The following morning, the membrane was washed three times for 10 min with 1× TBS-T. Secondary antibodies (anti-mouse or anti-rabbit, Jackson ImmunoResearch, West grove, PA, USA) were added at 1:5000 dilution in 2% I-Block in 1× TBS-T on a room temperature shaker for one hour. After the incubation, the membranes were washed three times for 10 min with 1× TBS-T. After the final wash, Luminol Reagent (Santa Cruz, Starr County, TX, USA) was added and allowed to react with the membranes for 2 min. The membranes were then developed using a FluorChem E Imager system (ProteinSimple, Santa Clara, CA, USA) exposing them for 1–30 min, depending on the intensity of the band. Densitometry was performed with the MCID image analysis program. The image density (1/intensity) of each band was normalized to β-actin, and experimental group band density was normalized to the band density of control [34].

### 4.7. Statistical Analysis

All measurements and analyses were performed by normality of distribution, and the homogeneity of variances was tested including the biochemistry and immunostaining. One-way analysis of variance (ANOVA), followed by a Tukey’s *post hoc* test, were performed for the immunostaining and Western blot analysis. All data are presented as mean ± standard error (SE).

## 5. Conclusions

Combination BMSCs and Niaspan treatment of stroke in diabetic rats increases the expression of CNPase, NG2, LFB, BS, Synaptophysin and SMI31 in the ischemic brain. Regulation of the above protein expression by combination treatment may promote white matter remodeling and thereby provide a beneficial effect after stroke in diabetic rats. Further investigations into the use of combination treatment as a therapeutic agent for the treatment of stroke in diabetics are warranted.
